# The Relationship Between Autistic Traits, Dating Violence and Anxiety in Adolescents

**DOI:** 10.1192/j.eurpsy.2023.1542

**Published:** 2023-07-19

**Authors:** M. Mutlu, P. Aydoğan Avşar, E. Çöp

**Affiliations:** Child and Adolescent Psychiatry, Ankara City Hospital, Ankara, Türkiye

## Abstract

**Introduction:**

Autistic traits that are continuously distributed in the population are characterized by difficulties in interpreting social information, deficits in understanding what others are thinking and feeling, difficulties in communicating ideas and emotions.

**Objectives:**

Otistic traits may elevate the risk for interpersonal victimization for those who exhibit them across the life course. In this study, the relationship between autistic traits, dating violence and anxiety levels were investigated in adolescents.

**Methods:**

The study included 61 adolescents aged 13-15 years and their parents who applied to the outpatient clinic for the first time and volunteered. Parents were asked to fill in the adolescent autism spectrum quotient (AQ) for their children, and the adolescent was asked to fill in the dating violence and screen for child anxiety related emotional disorders (SCARED) scales. It was hypothesized that adolescents that have higher AQ total scores have higher levels of dating violence and anxiety.

**Results:**

A total of 60 adolescents (44 girls and 16 boys) with a mean age of 14,6 were included in the study. A positive and significant correlation was found between autistic trait level and anxiety (r =.766, p = .00)and physical dating violence total scores (r =.259, p = .046). And also a positive and significant correlation was found between anxiety level and psychological (r =729, p = .00) and physical (r = .284, p = .028) dating violence total scores.

**Conclusions:**

In our study higher autistic traits were found to be associated with higher levels of anxiety and physical dating violence. Autistic traits in adolescents contribute to children’s anxiety level. Deficits in emotional and social cognition, inability to identify inappropriate behavior and one’s own discomfort with inappropriate behavior increase the risk of psychological and physical dating violence. Validated screening tools should be developed in this population to support earlier reporting.

**Disclosure of Interest:**

None Declared

